# Tracking the Influence of Thermal Expansion and Oxygen Vacancies on the Thermal Stability of Ni‐Rich Layered Cathode Materials

**DOI:** 10.1002/advs.201902413

**Published:** 2020-04-24

**Authors:** Eunkang Lee, Shoaib Muhammad, Taewhan Kim, Hyunchul Kim, Wontae Lee, Won‐Sub Yoon

**Affiliations:** ^1^ Department of Energy Science Sungkyunkwan University Suwon 440‐746 South Korea; ^2^ Materials Sciences Division Lawrence Berkeley National Laboratory Berkeley CA 94720 USA; ^3^ Present address: Department of Chemistry and Chemical Engineering Syed Babar Ali School of Science and Engineering (SBASSE) Lahore University of Management Sciences (LUMS) Lahore 54792 Pakistan

**Keywords:** Ni‐rich cathode materials, oxygen vacancy, thermal expansion, thermal stability

## Abstract

The ever‐growing demand for high‐energy lithium‐ion batteries in portable electronics and electric vehicles has triggered intensive research efforts over the past decade. An efficient strategy to boost the energy and power density of lithium‐ion batteries is to increase the Ni content in the cathode materials. However, a higher Ni content in the cathode materials gives rise to safety issues. Herein, thermal expansion and oxygen vacancies are proposed as new critical factors that affect the thermal stability of charged Ni‐rich cathode materials based on a systematic synchrotron‐based X‐ray study of Li_0.33_Ni_0.5+_ *_x_*Co_0.2_Mn_0.3‐_ *_x_*O_2_ (*x* = 0, 0.1, 0.2) cathode materials during a heating process. Charged cathode materials with higher Ni contents show larger thermal expansion, which accelerates transition metal migration to the Li layers. Oxygen vacancies are formed and accumulate mainly around Ni ions until the layered‐to‐spinel phase transition begins. The oxygen vacancies also facilitate transition metal migration to the Li layers. Thermal expansion and the presence of oxygen vacancies decrease the energy barrier for cation migration and facilitate the phase transitions in charged cathode materials during the heating process. These results provide valuable guidance for developing new cathode materials with improved safety characteristics.

## Graphical abstract

Close relationship between Ni content and inherent thermal instability of Ni‐rich cathode material has been widely investigated. However, the underlying cause of thermal instability of Ni‐rich cathode material involving Ni has not been fully studied. The thermal expansion and oxygen vacancy are newly proposed as critical factors to affect the inherent thermal instability of Ni‐rich cathode materials.

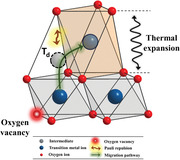

## 1 Introduction

Lithium‐ion batteries, the most successful electrochemical energy conversion and storage system thus far, have been utilized in electric vehicles and energy storage systems as a part of smart grids and portable electronics. Their widespread application has dramatically increased the demand for high‐capacity batteries. Ni‐rich cathode materials were introduced to achieve higher power and energy density in lithium‐ion batteries at lower cost.^[^ ^1^, ^2^, ^3^, ^4^, ^5^, ^6^ ^]^ However, the use of Ni‐rich cathode materials has led to serious safety problems in harsh thermal environments due to their inherent thermal instability.^[^ ^7^, ^8^, ^9^, ^10^ ^]^ The thermal runaway and catastrophic failure of lithium‐ion batteries are the result of the uncontrollable exothermic reactions, which mainly originate from the reaction between released oxygen and the electrolyte during the thermal decomposition of electrochemically charged cathode materials.^[^ ^11^, ^12^, ^13^, ^14^ ^]^ Studies on the thermal stability of Ni‐based cathode materials by thermogravimetric analysis, differential scanning calorimetry (DSC), and accelerating rate calorimetry show that the thermal decomposition reaction in charged cathodes starts at lower onset temperatures with increasing Ni content in the cathode materials, which indicates that a high Ni content worsens the thermal stability of the cathode material.^[^ ^15^, ^16^, ^17^, ^18^, ^19^ ^]^ Yoon et al. studied the thermal decomposition reaction of charged Ni‐rich cathode materials by in situ X‐ray diffraction (XRD) and specifically identified the phase transitions during heating.^[12]^ In combination with X‐ray techniques, density functional theory (DFT)‐based computational studies also contribute towards in‐depth understanding of thermal decomposition mechanism of layered cathode materials.^[^ ^20^, ^21^ ^]^ Nam et al. showed that local structure and oxidation state changes mainly occur around Ni in charged Ni‐based cathode materials during the heating process using the X‐ray absorption spectroscopy (XAS) technique.^[22]^ Moreover, the stable local environment of Mn ions directly contributes to the thermal stability of Ni‐based cathode materials by sustaining the MnO_6_ framework during the heating process. Additionally, changes in the electronic structure at the surface and in the bulk during the thermal decomposition reaction occur mainly around Ni, and thermal instability of the surface of charged Li_0.33_Ni_0.8_Co_0.15_Al_0.05_O_2_ cathode material was identified using the in situ soft XAS technique.^[23]^ The contribution of the Ni content to the thermal instability of charged Ni‐based cathode materials has been extensively investigated; however, the underlying cause of thermal instability involving Ni has not yet been fully explored. We focused on understanding the origin of the heat‐induced phase transitions involving Ni to obtain valuable guidance for designing safe and high energy batteries.

In this study, the thermal degradation mechanism of a series of Li_0.33_Ni_0.5+_ *_x_*Co_0.2_Mn_0.3‐_ *_x_*O_2_ (*x* = 0, 0.1, 0.2) cathode materials was systematically studied using synchrotron‐based characterization techniques, including in situ XRD, high‐resolution powder diffraction (HRPD) and XAS, combined with DSC. We observed, for the first time, that the thermal expansion in a charged cathode material is more intensive as the Ni content increases. Oxygen vacancies are formed and accumulate before the onset temperature of the phase transformation from the hexagonal layered to cubic spinel phase is reached. The detailed structural investigations in this study show that these specific thermal behaviors make cation migration energetically favorable and eventually facilitate the phase transition process. Thermal expansion and oxygen vacancy formation around Ni contribute to the initiation of the phase transition during the heating process. This study relates the thermal expansion and oxygen vacancy formation to the thermal instability of Ni‐rich cathode materials and explains the detailed thermal decomposition reaction in charged Li_0.33_Ni_0.5+_ *_x_*Co_0.2_Mn_0.3‐_ *_x_*O_2_ (*x* = 0, 0.1, 0.2) cathode materials.

## 2 Results and Discussion

**Figure 1:**
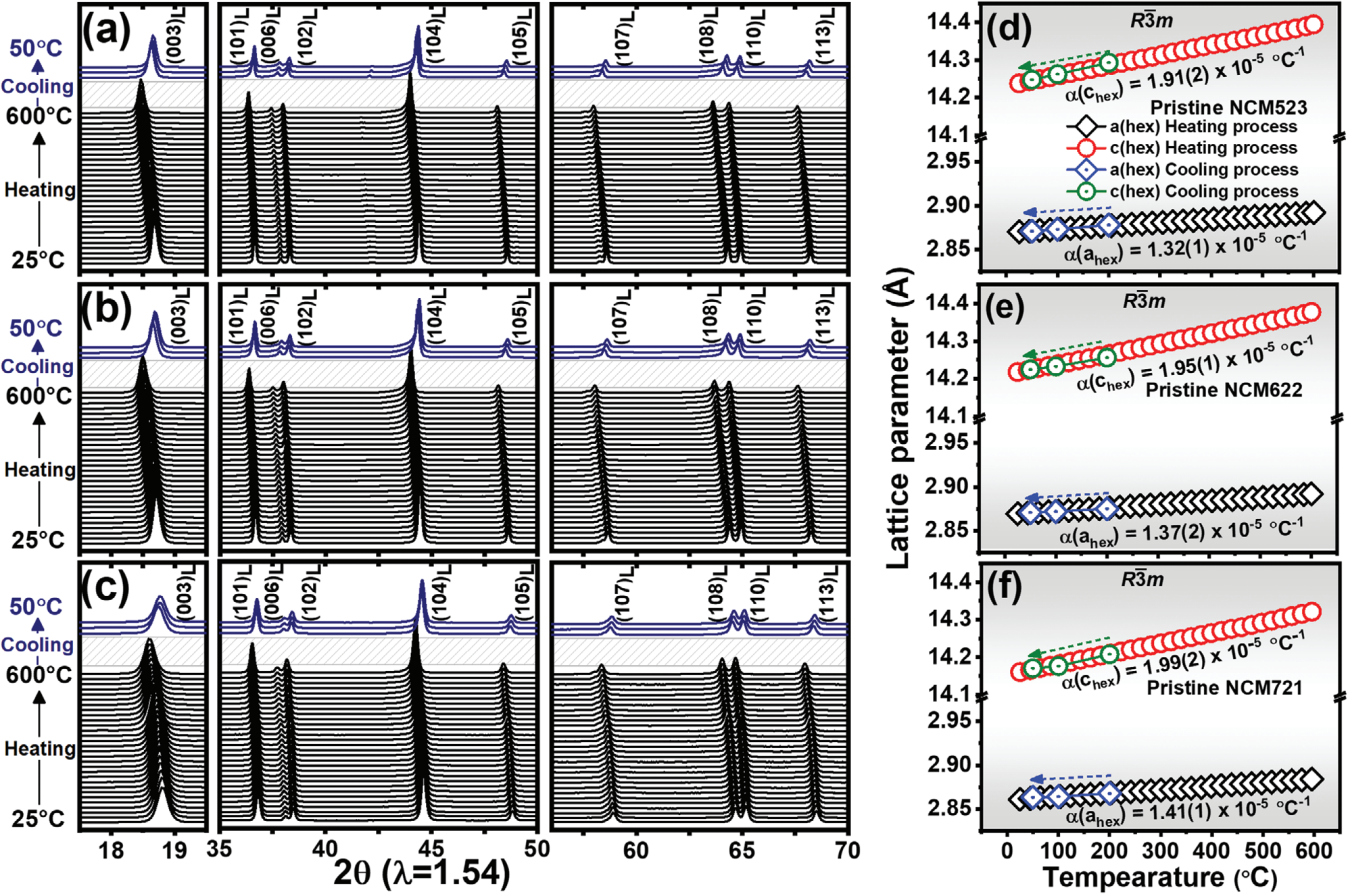
In situ XRD patterns and change in lattice parameters for pristine Ni‐rich cathode materials, a,d) LiNi_0.5_Co_0.2_Mn_0.3_O_2_, b,e) LiNi_0.6_Co_0.2_Mn_0.2_O_2_, and c,f) LiNi_0.7_Co_0.2_Mn_0.1_O_2_ during heating from 25 to 600 °C at a heating rate of 2.5 °C min^–1^ and cooling down to room temperature in the furnace. Subscript (L) represents hexagonal layered structure with $R\bar{3}m$ space group.

The heat‐induced thermal behavior of pristine LiNi_0.5+_ *_x_*Co_0.2_Mn_0.3‐_ *_x_*O_2_ (*x* = 0, 0.1, 0.2) cathode materials was investigated by measuring the in situ XRD patterns during heating from 25 to 600 °C and cooling to 50 °C. During cooling process, the furnace is turned‐off and the cathode materials are naturally cooled until 50 °C in the furnace. Heating rate is controlled while the cooling rate is not controlled inside the furnace. Stacked in situ XRD patterns of these cathode materials during heating and cooling are shown in **Figure** **1**a–c. Three cathode materials are shown as follows: LiNi_0.5_Co_0.2_Mn_0.3_O_2_ (NCM523), LiNi_0.6_Co_0.2_Mn_0.2_O_2_ (NCM622), and LiNi_0.7_Co_0.2_Mn_0.1_O_2_ (NCM721). The XRD patterns of these cathode materials can be indexed to a hexagonal layered phase with the $R\bar{3}m$ space group. The diffraction peaks shifted to lower 2*θ* values during heating, and returned to the initial positions after cooling, which indicates that the expansion and contraction of the LiNi_0.5+_ *_x_*Co_0.2_Mn_0.3‐_ *_x_*O_2_ (*x* = 0, 0.1, 0.2) crystal structure with the applied temperatures are reversible. To quantify the expansion and contraction, the lattice parameters of the hexagonal unit cell were calculated during the heating and cooling process. The calculated lattice parameters of NCM523, NCM622, and NCM721 through unit cell software using least squares fitting^[24]^ are shown in Figure 1d–f. The lattice parameter of these pristine cathode materials linearly increased during the heating process. This increase in the lattice parameters originates from the expansion of the unit cell in response to the increase in thermal vibrations of the lattice with increasing applied temperature.^[^ ^25^, ^26^ ^]^ When the temperature of the electrode materials returned to 50 °C after cooling process, the expanded lattices gradually shrunk and returned to their initial values. The slope of change in lattice parameters during the cooling process is similar to that during the heating process, which indicates that the rate of temperature change has no influence on the lattice parameter change. Furthermore, the change in lattice parameters quantitatively indicates that thermal expansion and contraction of the lattice in pristine LiNi_0.5+_ *_x_*Co_0.2_Mn_0.3‐_ *_x_*O_2_ (*x* = 0, 0.1, 0.2) cathode materials during heating and cooling are reversible. The coefficients of thermal expansion in *a*_hex._ and *c*_hex._ lattice parameter, *α*(*a*_hex._) and *α*(*c*_hex._), can be expressed as follows

(1) $$\begin{array}{c} \alpha \left(a_{\mathrm{hex}.}\right)=\;\frac{1}{a_{\mathrm{hex}.\mathrm{RT}}}\;\times \;\frac{da_{\mathrm{hex}.}}{dT} \end{array}$$

(2) $$\begin{array}{c} \alpha \left(c_{\mathrm{hex}.}\right)=\;\frac{1}{c_{\mathrm{hex}.\mathrm{RT}}}\;\times \;\frac{dc_{\mathrm{hex}.}}{dT} \end{array}$$

The *da*_hex._/d*T* and *dc*_hex._/d*T* are the rate of change of *a*_hex._ lattice parameter and *c*_hex._ lattice parameter per unit temperature change, respectively, and *a*_hex.RT_ and *c*_hex.RT_ are lattice parameter value at room temperature. The thermal expansion coefficients for the *a*_hex._ lattice parameter were 1.32(1) × 10^−5^, 1.37(2) × 10^−5^, and 1.41(1) × 10^−5^ °C^−1^ and the thermal expansion coefficients for the *c*_hex._ lattice parameter were 1.91(2) × 10^−5^, 1.95(1) × 10^−5^, and 1.99(2) × 10^−5^ °C^−1^ for NCM523, NCM622, and NCM721, respectively. The thermal expansion coefficients were different along the *a*_hex._ and *c*_hex._ axes in LiNi_0.5+_ *_x_*Co_0.2_Mn_0.3‐_ *_x_*O_2_ (*x* = 0, 0.1, 0.2) during heating. The layered cathode material consists of repetition of two layers of bond: weak Li─O and strong M─O (M = Ni, Mn, and Co) bond. The difference in the thermal expansion of the *a*_hex._ and *c*_hex._ lattice parameters is associated with the disparity of the weak Li─O and strong M─O bond strengths, and the distribution of the two kinds of bonds in the layered cathode material.^[^ ^27^, ^28^, ^29^ ^]^ The above thermal expansion coefficients of the pristine cathode materials show that the cathode material with higher Ni and lower Mn content, that is, NCM721, tends to be more sensitive to thermal expansion, which indicates that the Ni─O bond in this material is comparatively more responsive to heat than the Mn─O bond. The compositional difference in the Ni‐rich cathode materials not only changes their electrochemical characteristics but also affects the M─O interactions that play a significant role in the magnitude of the thermal expansion of materials under incident thermal energy.

**Figure 2:**
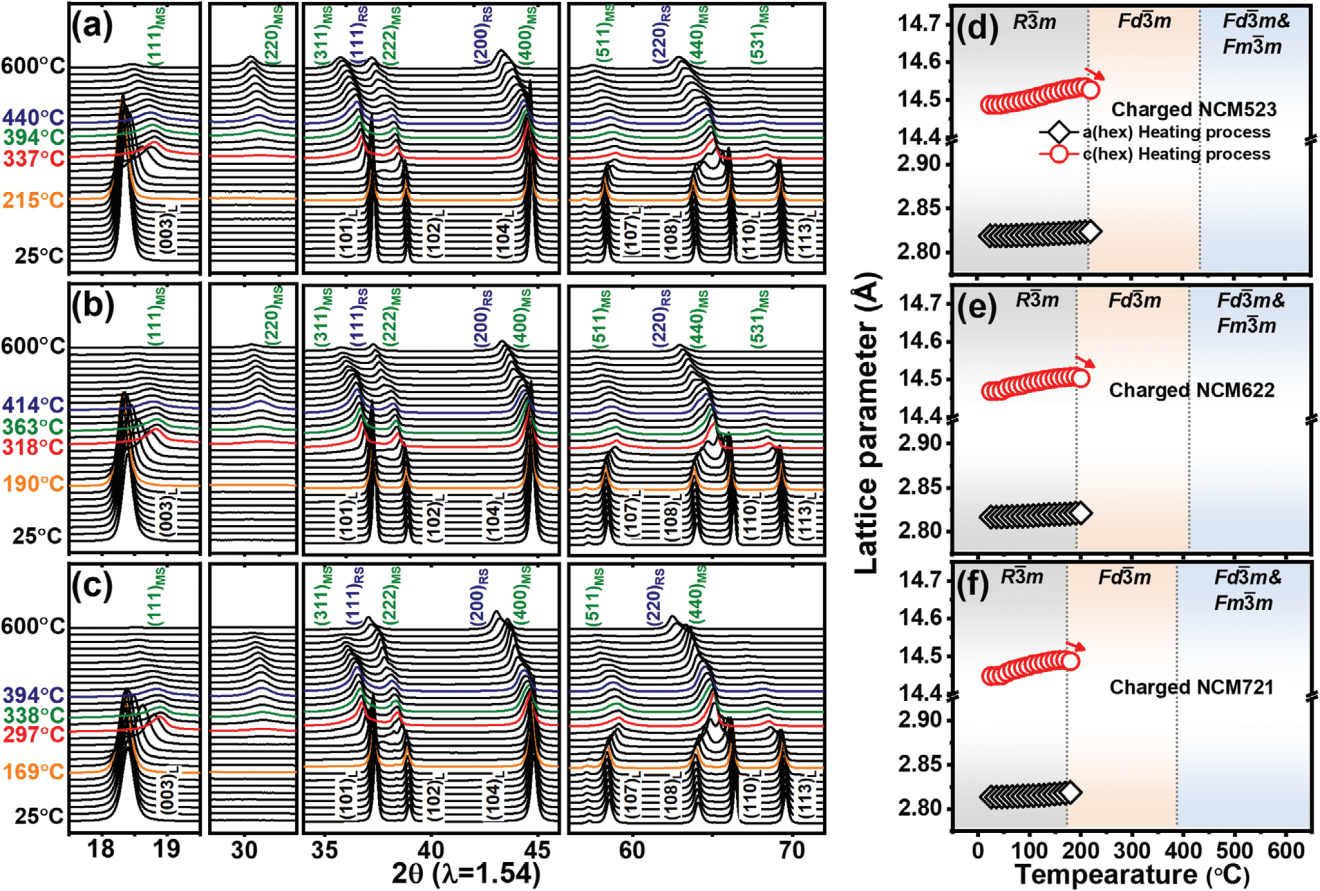
In situ XRD patterns and change in lattice parameters for the charged Ni‐rich cathode materials, a,d) Li_0.33_Ni_0.5_Co_0.2_Mn_0.3_O_2_, b,e) Li_0.33_Ni_0.6_Co_0.2_Mn_0.2_O_2_, and c,f) Li_0.33_Ni_0.7_Co_0.2_Mn_0.1_O_2_ during the heating from 25 to 600 °C at a heating rate of 2.5 °C min^–1^. Subscript (L) represents hexagonal layered structure, MS represents M_3_O_4_‐type spinel, and RS represents rock‐salt phase.

The thermal behavior of the charged Li_0.33_Ni_0.5+_ *_x_*Co_0.2_Mn_0.3‐_ *_x_*O_2_ (*x* = 0, 0.1, 0.2) cathode materials was studied by measuring the in situ XRD patterns during heating of these cathode materials from 25 to 600 °C. The stacked in situ XRD patterns of these cathode materials during the heating process are plotted in **Figure** **2**a–c. The thermal stability of the cathode materials depends on their state of charge,^[^ ^12^, ^30^ ^]^ and the initiation temperature of cation migration during a phase transition can be regulated by the Li content.^[31]^ To compare the thermal stabilities, LiNi_0.5+_ *_x_*Co_0.2_Mn_0.3‐_ *_x_*O_2_ (*x* = 0, 0.1, 0.2) cathode materials were charged to the same state of charge and their representative voltage profiles are shown in Figure S1, Supporting Information. At the start of the heating process, all peaks in the XRD patterns could be indexed to a hexagonal phase with the $R\bar{3}m$ space group and the diffraction peaks shifted to lower 2*θ* values during the initial heating, indicating thermal expansion. By further increasing the temperature of the charged cathode materials, the (108)_L_ and (110)_L_ peaks gradually merged into the (440)_S_ peak, and at the same time, the (003)_L_, (104)_L_, and (113)_L_ peaks that initially moved to lower 2*θ* values shifted to higher 2*θ* values, which indicates the transformation of the hexagonal $R\bar{3}m$ phase to a disordered cubic spinel $Fd\bar{3}m$ phase. The starting temperatures of this phase transition were 215, 190, and 169 °C for the charged NCM523, NCM622, and NCM721 cathode materials, respectively. The disordered cubic spinel phase is formed by cation migration in a hexagonal layered material; lithium ions move to the neighboring tetrahedral 8a sites and some of the transition metals (TMs) move to 16d octahedral sites.^[^ ^32^, ^33^ ^]^ The appearance of the (440)_S_ peak in the XRD patterns indicates the formation of a disordered spinel. The formation of the disordered spinel phase was completed at 337, 318, and 297 °C for charged NCM523, NCM622, and NCM721, respectively. Upon further heating of the charged cathode materials, the (220)_MS_ peak gradually appeared, which indicates further structural reordering of the disordered spinel phase. The appearance of the (220)_MS_ peak is a result of the transformation of the disordered spinel phase into an M_3_O_4_‐type spinel phase, in which some of the 8a tetrahedral sites as well as 16d octahedral sites are occupied by TM ions.^[^ ^34^, ^35^ ^]^ The formation temperatures of the M_3_O_4_‐type spinel phase were 394, 363, and 338 °C for the charged NCM523, NCM622, and NCM721 cathode materials, respectively. The continuous thermal decomposition reaction at high temperatures led to the formation of a disordered rock‐salt phase, which was reflected by the appearance of (111)_RS_, (200)_RS_, and (220)_RS_ peaks.^[9]^ The formation temperatures of the rock‐salt phase were 440, 414, and 394 °C for the charged NCM523, NCM622, and NCM721 cathode materials, respectively. The sequences of the heat‐induced phase transitions in the Li_0.33_Ni_0.5+_ *_x_*Co_0.2_Mn_0.3‐_ *_x_*O_2_ (*x* = 0, 0.1, 0.2) series were identical, but the onset temperatures of the phase transition were different. Equations (3) and (4) can be written by applying the above structure information of heat‐induced phase transition and Li content of the charged cathode materials to the previously proposed equations^[^ ^36^, ^37^, ^38^ ^]^

(3) $$\begin{array}{ccc} & & Li_{0.33}MO_{2}\to {\left[Li_{0.33}M_{0.113}\right]}_{8a}{\left[M_{0.886}\right]}_{16d}{\left[O_{1.773}\right]}_{32e}\left(Fd\bar{3}m\right)\;\\ & & \;+\;0.113O_{2} \end{array}$$

(4) $$\begin{array}{c} Li_{0.33}MO_{2}\to {\left[Li_{0.33}M\right]}_{4a}{\left[O_{1.33}\right]}_{4b}\left(Fm\bar{3}m\right)\;+0.335O_{2} \end{array}$$

The above equations show that oxygen release is involved in the heat‐induced phase transition process. For structural investigation of the detailed thermal decomposition reaction, the oxygen release from Ni‐rich cathode materials during heating and its effect on the phase transition process will be described in detail later in the XAS fitting results.

The lattice parameters of the Li_0.33_Ni_0.5+_ *_x_*Co_0.2_Mn_0.3‐_ *_x_*O_2_ (*x* = 0, 0.1, 0.2) cathode materials from 25 °C to the onset temperature of the phase transition from layered to disordered spinel were calculated through the unit cell software using least squares fitting for the in situ XRD patterns^[24]^ and are plotted in Figure 2d–f. The *a*_hex._ lattice parameter in the layered hexagonal structure mainly depends on the size of the MO_6_ octahedron, which is influenced by the average oxidation state of the TM ion.^[39]^ The *c*_hex._ lattice parameter is determined by the size of the LiO_6_ and MO_6_ octahedra. The trends of the *a*_hex._ and *c*_hex._ lattice parameters in the Li_0.33_Ni_0.5+_ *_x_*Co_0.2_Mn_0.3‐_ *_x_*O_2_ (*x* = 0, 0.1, 0.2) cathode materials during heating show a linear behavior in the temperature range before the onset of the phase transition, but the change of lattice parameters in the charged cathode materials is not as linear as the previously observed case of pristine LiNi_0.5+_ *_x_*Co_0.2_ Mn_0.3‐_ *_x_*O_2_ (*x* = 0, 0.1, 0.2) cathode materials. A drop in the *c*_hex._ lattice parameter is observed intermediately after the layered‐to‐disordered spinel phase transition. During the charge process of a Ni‐based layered cathode material, Li ions are extracted from the Li layers, and as a result, the *c*_hex._ lattice parameter expands due to the increase in the anionic repulsion between the oxygen layers across the Li layers.^[^ ^40^, ^41^ ^]^ A drop in the *c*_hex._ lattice parameter during heating indicates migration of TM ions to the Li layers during the phase transition from layered to disordered spinel since the screening effect due to this migration will decrease the repulsion between the oxygen layers.

**Figure 3:**
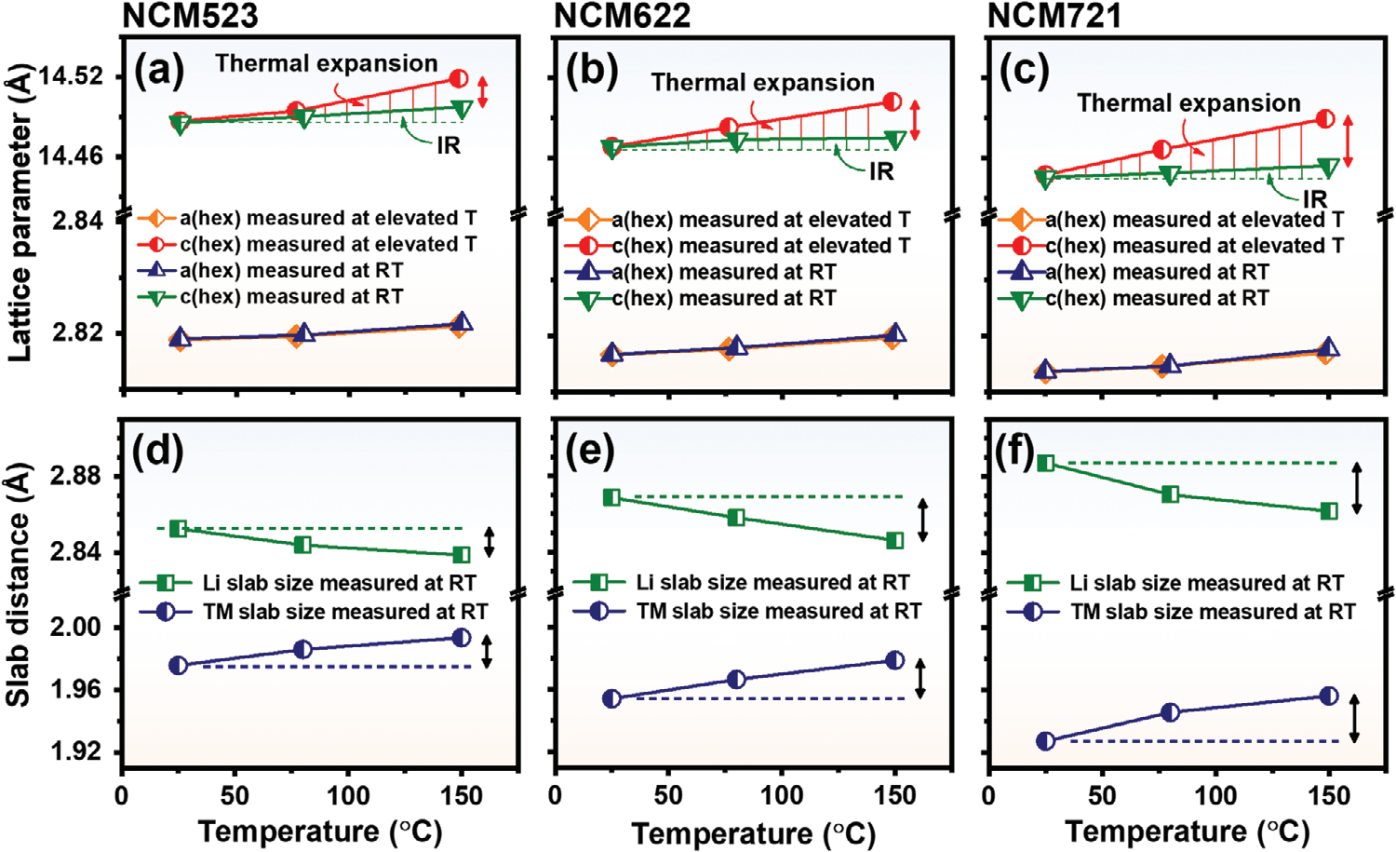
Changes in lattice parameters from the in situ XRD and HRPD refinement results are compared in (a–c). The areas with vertical red and green line indicate the increment of *c*_hex._ lattice parameter from thermal expansion and irreversible structural change (IR), respectively. The changes in slab size (d–f) are obtained from HRPD refinement results. The two experiments have different measurement conditions; the in situ XRD was measured at elevated temperature and HRPD experiment using heat‐treated charged Li_0.33_Ni_0.5+_ *_x_*Co_0.2_Mn_0.3‐_ *_x_*O_2_ (*x* = 0, 0.1, 0.2) cathode materials was measured at room temperature.

The pristine cathode materials show a linear change in the lattice parameters during heating to 600 °C without a phase transition; however, the change of lattice parameters in the charged cathode materials is not as linear as the change in the pristine cathode materials, even in the temperature range before onset of phase transition from layered to disordered spinel phase. For the charged Li_0.33_Ni_0.5+*x*_Co_0.2_Mn_0.3−*x*_O_2_ (*x* =0, 0.1, 0.2) cathode material prior to reaching the onset temperature of the phase transition, to identify the reversibility of thermal expansion and contraction confirmed in the pristine cathode materials, an HRPD experiment was conducted using the heat‐treated charged cathode materials which were cooled down to room temperature after heating to 80 and 150 °C. In this cooling process, the reversible structural change of the sample due to thermal expansion is omitted and only the irreversible structural change of the sample is maintained. These samples are termed heat‐treated samples in the following text. Rietveld refinement was performed on these heat‐treated materials.^[42]^ The detailed refinement results are shown in Table S1 and Figure S2, Supporting Information. A comparison of the lattice parameters at 25, 80, and 150 °C obtained from Rietveld refinement of the heat‐treated cathode materials and least squares fitting of the in situ XRD patterns of charged Li_0.33_Ni_0.5+_ *_x_*Co_0.2_Mn_0.3‐_ *_x_*O_2_ (*x* = 0, 0.1, 0.2) cathode materials is shown in **Figure** **3**a–c. The *y*‐axis scale ranges of *a*_hex._ and *c*_hex._ lattice parameter were equally set so that the difference between the maximum and minimum values is 1% of the minimum value, and the same absolute differences in each range of *a*_hex._ and *c*_hex._ lattice parameter plots represent the same percentage difference. The charged cathode materials used for HRPD and in situ XRD do not experience phase transition to disordered spinel phase in the compared temperature range from 25 to 150 °C. By increasing the temperature, the values of *a*_hex._ lattice parameters at each temperature in both measurement conditions (i.e., in situ XRD is measured during heating and HRPD experiment using heat‐treated sample is measured at room temperature) are relatively similar to each other, whereas the values of *c*_hex._ lattice parameters are not, which indicates that in these charged cathode materials, the changes in the *a*_hex._ lattice parameters are mainly driven by irreversible structural changes rather than reversible thermal expansion before the onset temperature of the phase transition is reached. The irreversible structural variations can derive from the reduction of the TM ions.^[^ ^39^, ^43^ ^]^ The interesting difference between the *c*_hex._ lattice parameters marked as red arrow in Figure 3a–c from in situ XRD and HRPD measurements arises mainly because of difference in measurement conditions between HRPD and in situ XRD techniques. The effects of both thermal lattice expansion and irreversible structural changes are included in in situ XRD. However, the effect of thermal lattice expansion is eliminated in HRPD results using heat‐treated samples that are cooled down to room temperature before measurement. Under this condition of HRPD, thermal lattice expansion is eliminated, and only irreversible structural changes can be studied. The interesting difference of charged NCM721 at 150 °C was about 80% of the increment of *c*_hex._ lattice parameters from 25 to 150 °C confirmed in the in situ XRD results. The remaining 20% corresponds to the irreversible structural change marked as IR in Figure 3a–c. The 80% was calculated from the information. In NCM721, the *c*_hex._ lattice parameters were 14.4457 Å (in situ XRD and HRPD) at 25 °C. At 150 °C, the *c*_hex._ lattice parameters were 14.4544 and 14.4892 Å from HRPD and in situ XRD, respectively. The thermal expansion accounted for major factor in *c*_hex._ lattice parameter change (80% in charged NCM721 at 150 °C) during heating. The interesting difference indicates the increment of *c*_hex._ lattice parameter by significant thermal expansion. The increment increased with increasing Ni content of the cathode material. The sizes of the Li and TM slabs calculated from the Rietveld refinement of the heat‐treated cathode materials are shown in Figure 3d–f. The TM slab size increased through reduction of TM ions in the MO_6_ octahedron and the Li slab size decreased in the heat‐treated samples with the increase in the temperature to 80 and 150 °C. These variations in slab size were more prominent in the charged cathode materials with higher Ni content. The change in the *c*_hex._ lattice parameter from the sum of the decrease in the Li slab size and the increase in the TM slab size was minor in the heat‐removed state, so the irreversible structural changes were not significantly reflected in the *c*_hex._ lattice parameter variations during heating due to the thermally reversible expansion. These findings show that during the heating process, the increase in the *a*_hex._ lattice parameter of the charged cathode material mainly derives from the reduction of the TM ions, and the increase in the *c*_hex._ lattice parameter mainly originates from the reversible thermal expansion before the onset temperature of the phase transition from the layered to disordered spinel phase is reached.

The significant reversible expansion of the *c*_hex._ lattice parameter can be divided into thermal expansions of the Li slab and TM slab. The coefficient of thermal expansion of the Li slab is more than three times that of the TM slab in pristine LiNi_0.5_Mn_0.5_O_2_.^[28]^ The difference in thermal expansion coefficient of TM slab and Li slab will be greater in a charged cathode material because the delithiation of Li results in the decrease in electrostatic force within the Li slab where Li ions interact with the two neighboring oxygen layers. The similar increase of the *a*_hex._ lattice parameter in different measurement conditions shows that the thermal expansion of *a*_hex._ lattice parameter is less in the charged sample. This indicates that the thermal expansion of the TM slab is also less than that of Li slab because the *a*_hex._ lattice parameter is very closely related to the size of TMO_6_ octahedron. These observations indicate that thermal expansion has a more pronounced effect on the Li slab compared to the TM slab and that the significant expansion of the *c*_hex._ lattice parameter originates from the prominent thermal expansion of the Li slab.

The bond angles and lengths in the LiO_6_ and TMO_6_ octahedrons were calculated from the Rietveld refinement results of the HRPD patterns of the charged and nonheated Li_0.33_Ni_0.5+_ *_x_*Co_0.2_Mn_0.3‐_ *_x_*O_2_ (*x* = 0, 0.1, 0.2) cathode materials. These results are shown in Figure S3 and Table S2, Supporting Information, and indicate that charged NCM721 has smaller O─Li─O and larger O─TM─O angles and has shorter TM─O and longer Li─O bonds, compared to charged NCM622 and NCM523. As the Ni content of the cathode material increases, the TMO_6_ octahedron of the charged cathode becomes more distorted in the form of smaller TM slab size and larger Li slab size. In charged cathode materials, the short Ni─O bonds bring the cations in the TM layer closer and increase the electrostatic repulsion between adjacent TM ions. As a result, it can be inferred that the octahedron is distorted to mitigate the electrostatic repulsion between TM ions. Also, the distortion trend according to Ni content of charged cathode is similar to that of pristine cathode.^[44]^ The charged cathodes with shorter Ni─O bonds are more distorted compared to distortions of pristine cathodes with longer Ni─O bonds. So, the size of Li slab is larger when the Ni content increases in charged cathode materials. Furthermore, the Ni^4+^─O bonds are present in large quantities in charged cathode materials with high Ni content, and the high covalent character of Ni^4+^─O bond reduces the effective oxygen charge, weakening the Li─O bonds.^[^ ^45^, ^46^ ^]^ Because the nonheated and charged cathode with higher Ni content has a longer and weaker Li─O ionic bond (i.e., the electrostatic force within the Li slab is weak), the Li slab in charged NCM721 is the most sensitive to thermal expansion among the Li_0.33_Ni_0.5+_ *_x_*Co_0.2_Mn_0.3‐_ *_x_*O_2_ (*x* = 0, 0.1, 0.2) cathode materials. Therefore, the thermal expansion of the Li slab in charged cathode materials is an inherent characteristic of the material depending on the Ni composition.

**Figure 4:**
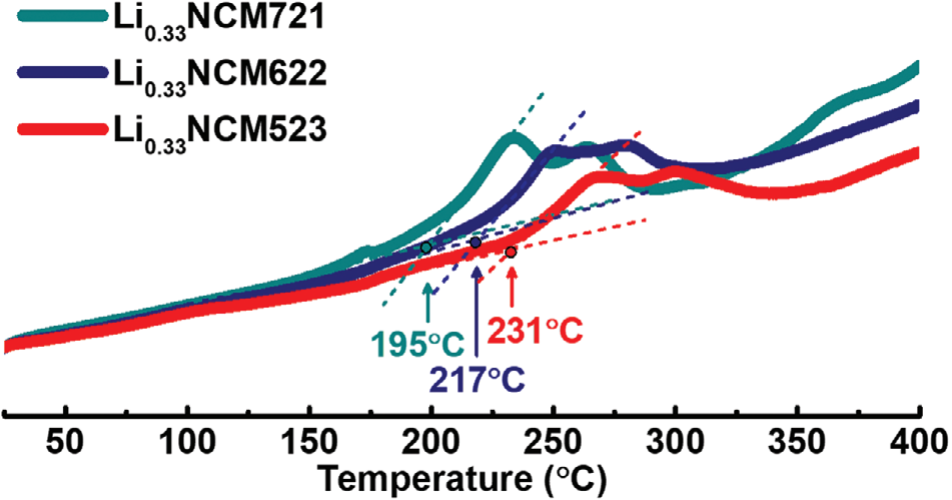
DSC curves for charged Li_0.33_Ni_0.5+_ *_x_*Co_0.2_Mn_0.3‐_ *_x_*O_2_ (*x* = 0, 0.1, 0.2) cathode materials in Ar gas filled pan at a heating rate of 2.5 °C min^–1^. The sample weights were equally 7.0 mg.

The relationship between the exothermic reaction and phase transition from layered to disordered spinel in charged Li_0.33_Ni_0.5+_ *_x_*Co_0.2_Mn_0.3‐_ *_x_*O_2_ (*x* = 0, 0.1, 0.2) cathode materials was investigated by DSC measurements. The DSC profiles are shown in **Figure** **4**, and the change in enthalpy and exothermic onset temperature calculated from the DSC profile are listed in **Table** **1**. Heat‐induced cation migration in hexagonal layered cathode materials is known to cause an exothermic reaction during the heating of the cathode material.^[36]^ The layered‐to‐spinel phase transition led by cation migration propagates from the surface to the bulk of the charged cathode material with increasing temperature.^[22]^ This gradual phase transition can result in broad exothermic peaks in the DSC profiles. The trends in the DSC profiles of the charged Li_0.33_Ni_0.5+_ *_x_*Co_0.2_Mn_0.3‐_ *_x_*O_2_ (*x* = 0, 0.1, 0.2) cathode materials show that the onset temperature for the exothermic reaction associated with the layered to spinel phase transition decreased with increasing Ni content in the charged cathode materials. This observation in the DSC profiles is consistent with the trends observed in the in situ XRD experiments; however, the phase transition temperatures in the DSC profiles compared to that of in situ XRD experiments are slightly different. This slight difference in onset temperatures can be attributed to the broad shape and low resolution of the exothermic peaks in the DSC profiles. The change in enthalpy (∆*H*) during the layered‐to‐disordered spinel phase transition increased in the cathode materials with higher nickel content, and the calculated changes in the enthalpy values were –236, –253, and –307 J g^–1^ for NCM523, NCM622, and NCM721, respectively. This trend of the change in enthalpy shows that the amount of heat released from the charged cathode material during the phase transition increased with increasing Ni content in the charged cathode materials. These observations from the change in enthalpy indicate that a higher Ni content in the layered cathode material facilitates cation migration and increases the heat released during the heat‐induced layered‐to‐spinel phase transition.

**Table 1:** Calculated change in enthalpy and exothermic onset temperature for charged Li_0.33_Ni_0.5+_ *_x_*Co_0.2_Mn_0.3‐_ *_x_*O_2_ (*x* = 0, 0.1, 0.2) cathode materials is obtained from DSC curves as shown in Figure 4. Starting temperatures of layered‐to‐disordered spinel phase transition are extracted from the results of in situ XRD as shown in Figure 2

	NCM523	NCM622	NCM721
Enthalpy [Δ*H*]	–236 J g^–1^	–253 J g^–1^	–307 J g^–1^
Exothermic onset T	231 °C	217 °C	195 °C
Starting T to disordered spinel	215 °C	190 °C	169 °C

**Figure 5:**
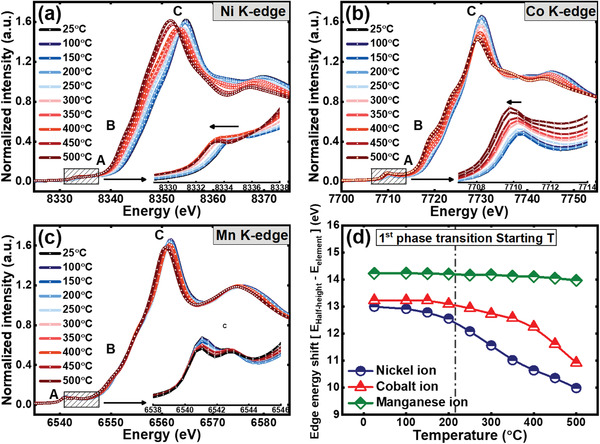
Normalized a) Ni K‐edge, b) Co K‐edge, and c) Mn K‐edge XANES spectra and change in difference between half‐height energy and edge energy of metal as function of heating temperature (d) for charged Li_0.33_Ni_0.5_Co_0.2_Mn_0.3_O_2_ cathode material after heating at various temperatures. The dashed line indicates the starting temperature of phase transition from layered to disordered spinel phase.

Since the thermal stability of the Li_0.33_Ni_0.5+_ *_x_*Co_0.2_Mn_0.3‐_ *_x_*O_2_ (*x* = 0, 0.1, 0.2) cathode materials is influenced by the proportion of Ni, XAS can be an ideal technique to specifically study the contribution of each TM ions to the thermal decomposition reaction of these cathode materials. The XAS spectrum consists of X‐ray absorption near edge structure (XANES), which contains information on the oxidation state and coordination geometry of the absorbing atom, and extended X‐ray absorption fine structure (EXAFS), which can be used to evaluate the local structure around the absorbing atom. The Ni, Co, and Mn K‐edge XANES spectra of the charged Li_0.33_Ni_0.5_Co_0.2_Mn_0.3_O_2_ cathode material heated to a temperature between 25 and 500 °C are plotted in **Figure** **5**a–c. The weak absorption pre‐edge peak, labeled “A” in the XANES spectra, is associated with a forbidden electronic transition from a core 1s electron to the 3d orbital. The hybridization of the 3d and 4p orbitals due to structural distortions in the local symmetry between the TM and oxygen partially enables this dipole‐forbidden electronic transition, resulting in a weak pre‐edge peak.^[47]^ The shoulder peak and strong main absorption peak marked “B” and “C” arise due to the dipole‐allowed electronic transition of a core electron in the 1s orbital to an unoccupied 4p orbital bound state with T_1u_ symmetry. The electronic transition of shoulder peak is associated with the ligand‐to‐metal charge transfer effect, which can be regulated by the oxidation state of the absorbing atom due to the difference in the numbers of outermost electrons.^[^ ^47^, ^48^ ^]^ Figure 5d shows the change in the absorption edge energy shift of XANES at the Ni, Co, and Mn K‐edges, calculated from the difference between the edge energy of each metal and the half‐height energy position of the XANES spectra. A prominent shift in the energy position of the Ni K‐edge XANES spectra is observed, and the energy position of the Co K‐edge XANES spectra shows a smaller shift than that of the Ni K‐edge as the temperature increases. The Mn K‐edge XANES spectra show some minor changes, but these variations can be disregarded because no change in the overall edge energy position occurred.^[49]^ These minor changes in the Mn K‐edge XANES spectra are often referred as variations in the local structure induced by changes in the oxidation state of neighboring TM ions such as Ni.^[50]^ The XANES spectra show that a prominent reduction of Ni^4+^, a slight reduction of Co^3+^, and almost no change in the Mn^4+^ oxidation state occurred in the heat‐treated Li_0.33_Ni_0.5_Co_0.2_Mn_0.3_O_2_ cathode materials in the range of 25 to 300 °C. Interestingly, a modest reduction of Ni started even below the onset temperature of the layered‐to‐spinel phase transition, unlike the Co and Mn ions. These observations from the XANES spectra confirm that Ni is the most thermally unstable ion in the charged Li_0.33_Ni_0.5_Co_0.2_Mn_0.3_O_2_ cathode material whereas Mn is the most thermally stable ion in this cathode material.

**Figure 6:**
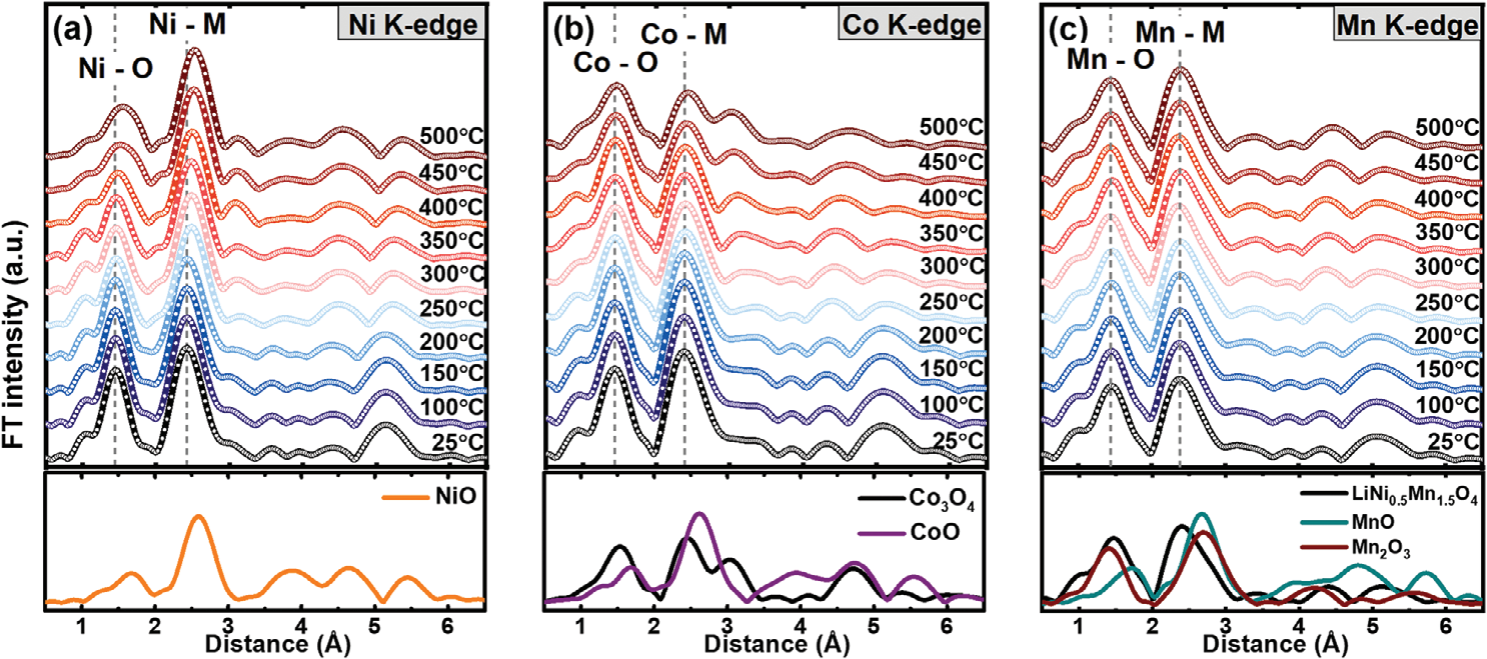
Comparison of the a) Ni, b) Co, and c) Mn K‐edge Fourier transform magnitudes of *k* ^3^‐weighted EXAFS spectra of charged Li_0.33_Ni_0.5_Co_0.2_Mn_0.3_O cathode material after heating in the range of several different temperatures up to 500 °C. The spectra shown below are reference spectra for corresponding TM oxide.

To understand the local structural variations in the charged Li_0.33_Ni_0.5_Co_0.2_Mn_0.3_O_2_ cathode material after heating from 25 to 500 °C, *k* ^3^‐weighted Ni, Co, and Mn K‐edge EXAFS spectra are plotted in **Figure** **6**a–c. Two prominent peaks is observed in each of these EXAFS spectra; the first peak represents the M─O (M = Ni, Co, and Mn) bond interactions and the second peak is associated with the M─M interactions. The peak positions in the EXAFS spectrum can be associated with the bond lengths in the M─O or M─M coordination shells, respectively. The first and second peak features in the Ni and Co K‐edge spectra changed with increasing heat treatment temperature, indicating local structural variation around Ni and Co; however, no significant changes occurred in the Mn K‐edge EXAFS spectra. The intensities of the Ni─O and Co─O peaks dropped as the heat treatment temperature increased. By comparing the Co_3_O_4_ and NiO reference EXAFS spectra, when heating the charged Li_0.33_Ni_0.5_Co_0.2_Mn_0.3_O_2_ cathode material beyond 400 °C, M_3_O_4_‐type spinel and MO‐type disordered rock‐salt phases can be inferred to mainly form around Co and Ni ions, respectively. However, the first and second FT spectra peaks of the Mn K‐edge were almost constant over the entire temperature range. These results indicate that the local structure around Mn ions is more thermally stable than those around Ni and Co ions.

**Figure 7:**
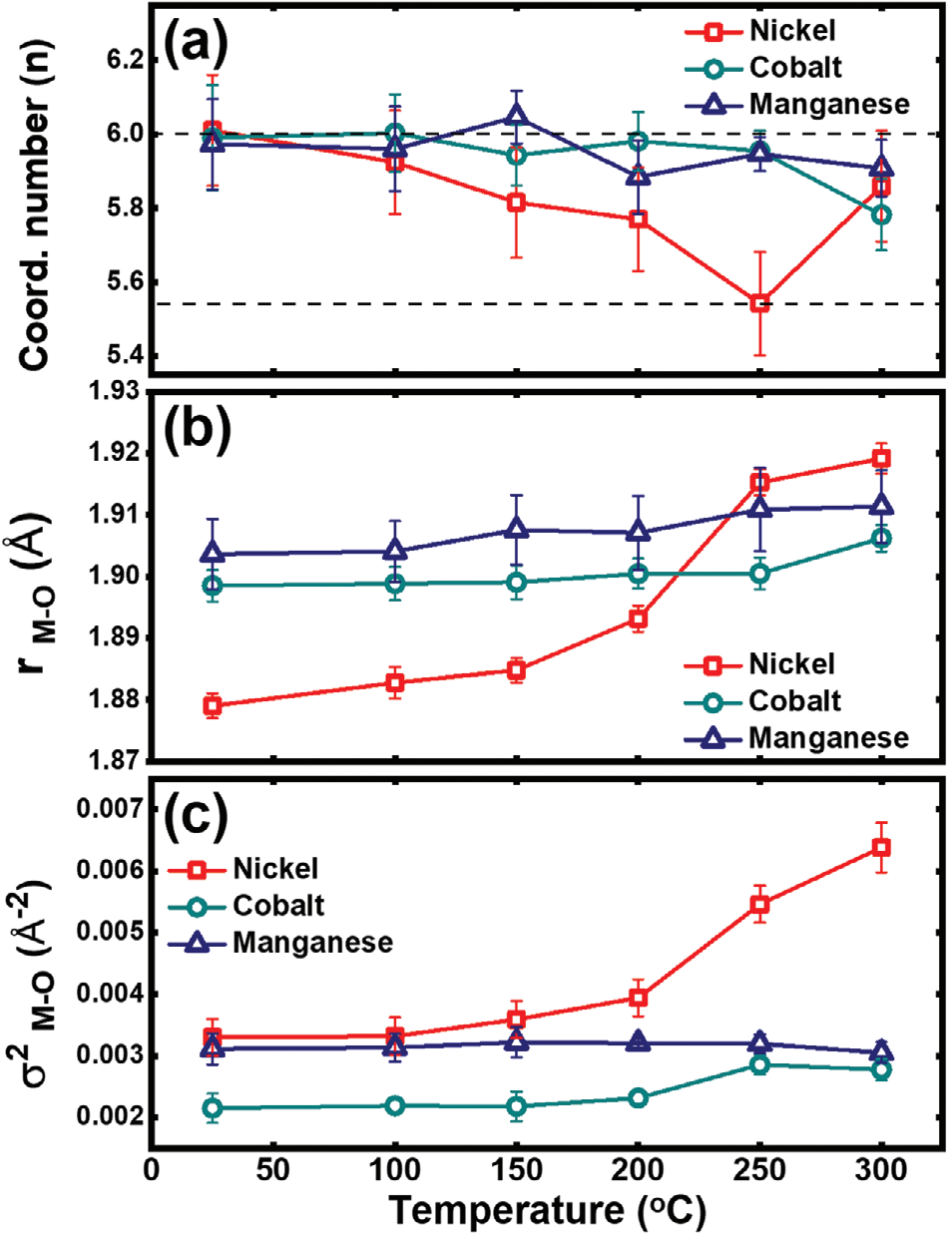
a) Coordination numbers, b) bond lengths, and c) Debye–Waller factors (c) of first coordination shell (MO_6_ octahedron) are results by *k*‐weight (*k* = 1, 2, 3)‐dependent fitting using $R\bar{3}m$ space group on Ni, Co, and Mn K‐edge EXAFS spectra of Li_0.33_Ni_0.5_Co_0.2_Mn_0.3_O_2_ cathode material in the range of various temperatures up to 300 °C. For the accuracy of the result, the fitting was performed only up to 300 °C because the Li_0.33_Ni_0.5_Co_0.2_Mn_0.3_O_2_ cathode material is transformed to the disordered cubic spinel structure above 350 °C.

Fitting of the EXAFS spectra was performed to observe the variations in the M─O bond length and coordination number along with the Debye–Waller factor to understand the specific changes in the local structure of the Li_0.33_Ni_0.5_Co_0.2_Mn_0.3_O_2_ cathode material as the heat treatment temperature increased. The coordination number fitting of EXAFS spectra has an error margin of ≈20% because of the strong correlation between the Debye–Waller factor and the coordination number on the Fourier transform intensity.^[^ ^51^, ^52^ ^]^ This correlation can be minimized by performing a *k*‐weight (*k* = 1, 2, 3)‐dependent fitting on the first coordination (M─O) shell.^[^ ^53^, ^54^ ^]^ *k*‐Weight (*k* = 1, 2, 3)‐dependent fitting of the Ni, Co, and Mn K‐edge EXAFS spectra of the heat‐treated and charged Li_0.33_Ni_0.5_Co_0.2_Mn_0.3_O_2_ cathode materials from 25 to 300 °C was performed. The fitting results are shown in **Figure** **7**, and the fitting details are provided in Figures S4 and S5, Supporting Information. The fitting results show that the Ni─O bond length significantly increased with increasing temperature, indicating reduction of Ni^4+^, and almost no change occurred in the Co─O and Mn─O bond lengths in this temperature range. In stoichiometric Li_0.33_Ni_0.5_Co_0.2_Mn_0.3_O_2_, Ni, Co, and Mn are coordinated with six oxygen atoms; however, the Ni─O coordination number drops to ≈5.5 upon heating to 250 °C and then recovers to ≈5.8 upon further heating to 300 °C. A small drop occurs in the Co─O coordination number, while that of Mn─O remains almost unchanged. The drop in the Ni─O coordination number from 25 to 250 °C indicates that oxygen vacancies are created and accumulate around Ni. The main phase of charged cathode material changes from hexagonal layered to disordered cubic spinel at temperature around 300 °C as shown in Figure 2a. This crystal structure rearrangement helps to recover the Ni─O coordination number to ≈5.8 at 300 °C and decreases the number of oxygen vacancies. The occurrence of oxygen vacancies and reduction of Ni can be supported by the electronic paramagnetic resonance (EPR) results as shown in Figure S6, Supporting Information. The formation and disappearance of oxygen vacancies around Ni in this temperature range is involved in the oxygen release during the hexagonal layered‐to‐disordered spinel phase transformation as described in Equation (3). This oxygen release can influence the shrinkage of size of the Li slab with increasing heat treatment temperature as shown in Figure 3d–f, since oxygen release can reduce the oxygen‐oxygen anionic repulsion by directly removing the anion itself. The Debye–Waller factor is an indicator of the distortion of the crystal structure. The formation of Ni^3+^, a Jahn–Teller active ion, as a result of the reduction of Ni^4+^ ions and formation of oxygen vacancies around Ni ions significantly increases the disorder of the crystal structure beyond 200 °C.

Generally, the changes in the oxidation states of Co and Mn are relatively consistent compared to that of Ni during the electrochemical charge process of LiNi_1‐_ *_x_*_‐_ *_y_*Co*_x_*Mn*_y_*O_2_ cathode materials.^[^ ^55^, ^56^, ^57^ ^]^ Assuming that the oxidation states of Co and Mn ions are maintained at 3+ and 4+, respectively, during the charge process, the cathode material with a higher Ni content will include a larger amount of Ni^4+^, as shown in **Table** **2**. Based on the DFT calculations, a large amount of Ni^4+^ ions is formed in charged cathode materials with a high Ni content, and the Ni^4+^ ions can be easily reduced by obtaining electrons from oxygen ions due to the high covalent character of Ni^4+^─O.^[46]^ The main reduction of Ni^4+^ ions leads to the formation of oxygen vacancies around Ni. Therefore, the formation of oxygen vacancies around Ni can be inferred to be more pronounced in a cathode material with a higher Ni content.

**Table 2:** Assuming that the net charge of the material is zero and that the oxidation state of cobalt and manganese ions is fixed in tri‐ and tetra‐state, respectively, the contents of Ni^3+^ and Ni^4+^ ion in charged Ni‐rich cathode materials can be calculated using simple simultaneous equations. The starting temperatures to spinel are based on the results of in situ XRD

	NCM523	NCM622	NCM721
Average oxidation state of nickel ion	3.740	3.783	3.814
Content of Ni^3+^ ion (atomic ratio)	0.13	0.13	0.13
Content of Ni^4+^ ion (atomic ratio)	0.37	0.47	0.57
Starting temperature to disordered spinel	215 °C	190 °C	169 °C

**Figure 8:**
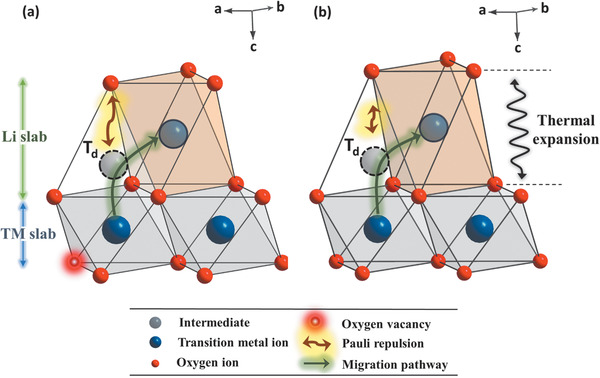
Formation of oxygen vacancies in MO_6_ octahedron makes the cation migration through face‐shared oxygen plane without oxygen vacancy energetically favorable. The energetically mitigated pathway for migration of TM ion from octahedral site in TM layer to octahedral site in Li layer is shown in (a). Expanded pathway due to the thermal expansion on Li slab (b) provides area with less Pauli repulsion between oxygen ion and migrating cation, which allows migrating cation to move more freely. Red spot with white center indicates the oxygen vacancy and hollow dot black circle represents the tetrahedral site (*T*_d_). The hollow black circle in Li slab is the octahedral site that migrating cation can take after cation migration.

In the process of the transformation from the hexagonal layered to disordered spinel phase, TM ions migrate from the TM layers to empty Li sites in the Li layer by hopping through neighboring tetrahedral sites (*T*_d_). In this case, the cations migrate through the planes instead of the corners and edge of the octahedra due to the strong Pauli repulsion between oxygen and migrating cation.^[58]^ In the presence of oxygen vacancies around Ni, more space is available for cation migration due to the reduction in the Pauli repulsion between migrating cations and oxygen ions. Migrating cations may pass through the oxygen vacancies. However, the strong Coulomb repulsion between the migrating cations and neighboring cations in the TM layer raises the energy barrier for cation migration through oxygen vacancies.^[59]^ When oxygen vacancies are present in the oxygen framework, cation migration through the intermediate *T*_d_ must be considered a major pathway. The previous theoretical work suggests that when oxygen vacancies are formed in the MO_6_ octahedra of a lithium‐excess layered oxide, the migrating cations prefer to move through the face‐shared oxygen plane without oxygen vacancies due to the low energy barrier for cation migration, although the Pauli repulsion effect is lower for the migration through the face‐shared oxygen plane with oxygen vacancies.^[60]^ The positive charge (Kröger–Vink notation $V_{O}^{••}$) of oxygen vacancies can inhibit cation migration through the face‐shared oxygen plane with oxygen vacancies due to the Coulomb repulsion between the migrating cations and oxygen vacancies. This variation in the energy barrier for cation migration with the position of the oxygen vacancies was also observed in the LiNiO_2_ layered cathode material.^[61]^ Considering the formation of oxygen vacancies around Ni, the cations will migrate through the face‐shared oxygen plane that does not contain oxygen vacancies during heating as shown in **Figure** **8**a, because the energy barrier in this migration pathway is more energetically favorable than that in the pathway without oxygen vacancies, the pathway through oxygen vacancies, and the pathway through the face‐shared oxygen plane with oxygen vacancies. The formation of oxygen vacancies provides the pathway with a low energy barrier and facilitates the cation migration during the heating process. Additionally, the charged cathode material with a higher Ni content exhibits a larger thermal expansion of the Li slab during heating, which expands the pathways for cation migration as shown in Figure 8b. The size of intermediate tetrahedron present in the cation migration pathway is mainly regulated by the size of Li slab. When the TM cation crosses the tetrahedron, the elongation of the intermediate tetrahedron in the *c*‐axis direction by thermal expansion of the Li slab increases the distance between the surrounding oxygen and the migrating cation, thereby lowering the Pauli repulsion. The thermal expansion further lowers the onset temperature of the phase transition by decreasing the energy barrier for cation migration. Therefore, the oxygen vacancies and thermal expansion, which can regulate the energy barrier for cation migration, in a charged Ni‐rich cathode material are considered major factors that explain how a high Ni content induces thermal instability of a layered cathode material.

Based on the above discussion, on the effect of thermal expansion and oxygen vacancy formation around Ni, the phase transition during heating can be summarized as follows. When the temperature applied to the charged Li_0.33_Ni_0.5+_ *_x_*Co_0.2_Mn_0.3‐_ *_x_*O_2_ (*x* = 0, 0.1, 0.2) cathode material increases beyond 25 °C, thermal expansion of the crystal structure is observed. This thermal expansion is more pronounced for the *c*_hex._ lattice parameter than for the *a*_hex._ lattice parameter, specifically in the Li slabs, and broadens the cation migration pathways. At the same time, oxygen vacancies are formed and accumulate mainly around Ni. In the presence of these vacancies, the cation migration pathway in which the migrating cations move through the face‐shared oxygen plane that does not contain oxygen vacancies is energetically mitigated. This pathway, under the effects of thermal expansion and oxygen vacancies, has a lower energy barrier for cation migration. When the Ni ions located at the octahedral sites in the TM layer have sufficient energy for cation migration during heating, some of the Ni ions mainly migrate to empty octahedral sites in the Li layers, which induces the hexagonal layered‐to‐disordered spinel phase transition in the charged cathode material. The migration of Ni is facilitated by the presence of oxygen vacancies and thermal expansion. However, the migration of Co and Mn is facilitated only by lowering of the energy barrier due to thermal expansion since oxygen vacancies are rarely observed around Co and Mn. Upon heating beyond the phase transition temperature from layered to disordered spinel, phase transitions continue, and the disordered spinel phase gradually transforms to an M_3_O_4_‐type ordered spinel phase around Co. EXAFS spectra show that the MO‐type rock‐salt phase involving Ni is formed when heating beyond 400 °C. This study demonstrates for the first time, the critical role of the Ni content in determining the energy barrier for cation migration and the onset temperature of the thermal decomposition reaction in charged Ni‐rich cathode materials. In cathode materials with a high Ni content, the thermal expansion magnitude is significantly large, and oxygen vacancy formation can be promoted. These factors accelerate the thermal decomposition reaction and eventually result in thermal instability of the Ni‐rich cathode material.

## 3 Conclusion

In this study, the thermal decomposition process of charged Li_0.33_Ni_0.5+_ *_x_*Co_0.2_Mn_0.3‐_ *_x_*O_2_ (*x* = 0, 0.1, 0.2) cathode materials was systematically investigated using a combination of synchrotron‐based XRD, XAS, and a thermoanalytical DSC technique. From the XRD results of the charged cathodes during the heating process, the charged cathode material with a higher Ni content exhibits a larger thermal expansion during the heating process. The XAS results showed that before the onset temperature of the layered‐to‐spinel phase transition is reached, the Ni ions are reduced, which is accompanied by the formation of oxygen vacancies around Ni ions. The thermal expansion and oxygen vacancy formation in charged Ni‐rich cathode materials facilitate TM migration and lower the onset temperature of the thermal decomposition reaction by activating an energetically favorable pathway for cation migration. Therefore, thermal expansion and oxygen vacancy formation should be considered new critical factors that affect the thermal stability of charged Ni‐rich cathode materials. These findings can provide a better understanding of the inherent thermal instability of Ni‐rich cathode materials, thereby helping design safer Ni‐rich cathode materials for next generation Li rechargeable batteries.

## 4 Experimental Section

A series of LiNi_0.5+_ *_x_*Co_0.2_Mn_0.3‐_ *_x_*O_2_ (*x* = 0, 0.1, 0.2) cathode materials were synthesized by a coprecipitation method. Aqueous solutions with stoichiometric amounts of precursors (NiSO_4_·7H_2_O/CoSO_4_·7H_2_O/MnSO_4_·H_2_O = 5:2:3, 6:2:2, and 7:2:1) were stirred in 1.5 M NaOH solution at 50 °C and a pH of 11. After the coprecipitation reaction, the products were washed with deionized water and dried at 120 °C for 24 h. The products were mixed with a lithium source (LiOH) at a fixed lithium‐to‐metal ratio of 1.02:1, and the mixtures were calcined at 850 °C for 12 h in an air environment. Scanning electron microscopy images in Figure S7, Supporting Information, show that three well‐synthesized LiNi_0.5+_ *_x_*Co_0.2_Mn_0.3‐_ *_x_*O_2_ (*x* = 0, 0.1, 0.2) cathode powders consist of the spherical secondary particles densely packed by primary particles and have a homogeneous morphology. The primary and secondary particles each have a similar size in three LiNi_0.5+_ *_x_*Co_0.2_Mn_0.3‐_ *_x_*O_2_ (*x* = 0, 0.1, 0.2) cathode powders.

Cathode slurries with active material (92 wt%), carbon black (4 wt%), and a polyvinylidene fluoride (PVDF) binder (4 wt%) were coated on Al foils and then dried at 120 °C for 12 h in vacuum. These cathodes were assembled into 2032‐type coin cells with a Li metal anode, a Celgard separator, and an electrolyte (1.15 M LiPF_6_ dissolved in a 3:7 ethyl carbonate/dimethyl carbonate solvent with 2% vinylene carbonate) in an argon‐filled glove box. Electrode samples for X‐ray studies were prepared by disassembling the coin cells in an Ar‐filled glove box and washing the working electrodes with anhydrous DEC.

In situ XRD measurements were performed during heating of the powder from 25 to 600 °C at a heating rate of 2.5 °C min^–1^. For in situ XRD measurements of charged cathode materials, electrodes were scraped to separate the cathode powders from the Al foil. The powdered sample contains active cathode material, carbon black, and PVDF binder. For DSC measurements on charged cathode materials, the charged powders were sealed inside Al pan inside the Ar‐filled glove box, and Al pan was mounted on the diffraction system during DSC measurements. HRPD patterns of the electrode samples heated to 25, 80, and 150 °C were measured. The heat‐treated samples were cooled down to room temperature before HRPD measurement. The incident X‐rays were vertically collimated using a mirror and monochromatized to a wavelength of 1.5176 Å, using a double‐crystal Si (111) monochromator. The detector arm of the diffractometer had Soller slits with an angular resolution of 2°, a flat Ge (111) crystal analyzer, an antiscatter baffle and seven scintillation detectors. XAS spectra were collected in transmission mode using ionization chambers and a Si (111) double‐crystal monochromator detuned to ≈80% of its original intensity to eliminate higher order harmonics. Data preprocessing operations such as deglitching, energy calibration, normalization, and least square fitting with theory were performed as described by Kelly et al.^[62]^ using IFEFFIT^[63]^ which uses the FEFF code.^[64]^ DSC measurements were performed with a Seiko DSC 7020 (THASS, Germany) at a heating rate of 2.5 °C min^–1^ up to 400 °C using an Ar‐filled pan that is sealed with charged cathode material (including the binder and conductive carbon).

## Conflict of Interest

The authors declare no conflict of interest.

## Supporting information

Supporting Information
