# Supplementary material for: Tracking the Influence of Thermal Expansion and Oxygen Vacancies on the Thermal Stability of Ni‐Rich Layered Cathode Materials
Source: Adv Sci (Weinh). 2020 Apr 24;7(12):1902413. doi: 10.1002/advs.201902413 (PMC7312338; doi:10.1002/advs.201902413)
Supplement: Supplementary file 1 — Supporting Information [file ADVS-7-1902413-s001.pdf]

Copyright WILEY-VCH Verlag GmbH & Co. KGaA, 69469 Weinheim, Germany, 2018.

Supporting Information

Title : Tracking Influence of Thermal Expansion and Oxygen Vacancy on Thermal Stability of Ni-rich Layered Cathode Materials

*Eunkang Lee, Shoaib Muhammad, Taewhan Kim, Hyunchul Kim, Wontae Lee, Won-Sub Yoon**

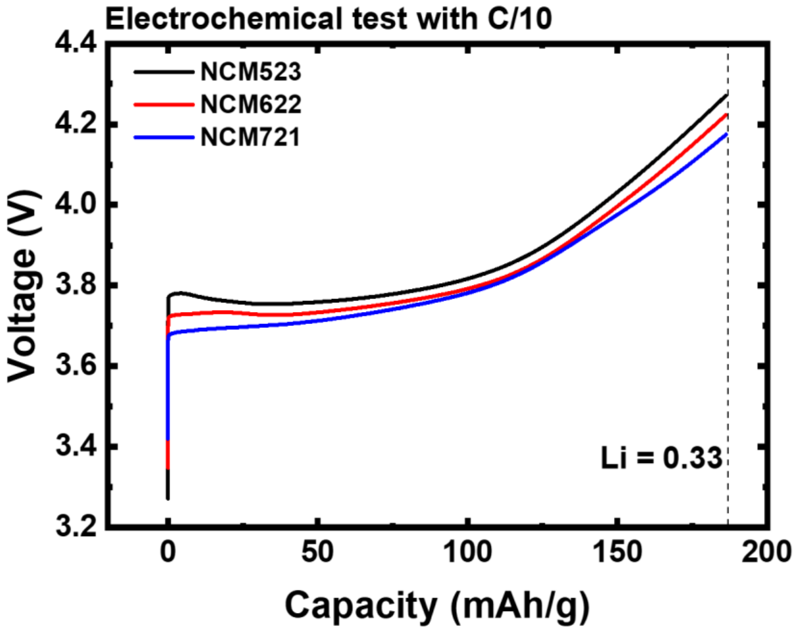

Figure S1. The first charge voltage profile of $Li_{0.33}Ni_{0.5}Co_{0.2}Mn_{0.3}O_2$ (NCM523), $Li_{0.33}Ni_{0.6}Co_{0.2}Mn_{0.2}O_2$ (NCM622), and $Li_{0.33}Ni_{0.7}Co_{0.2}Mn_{0.1}O_2$ (NCM721) for C/10.

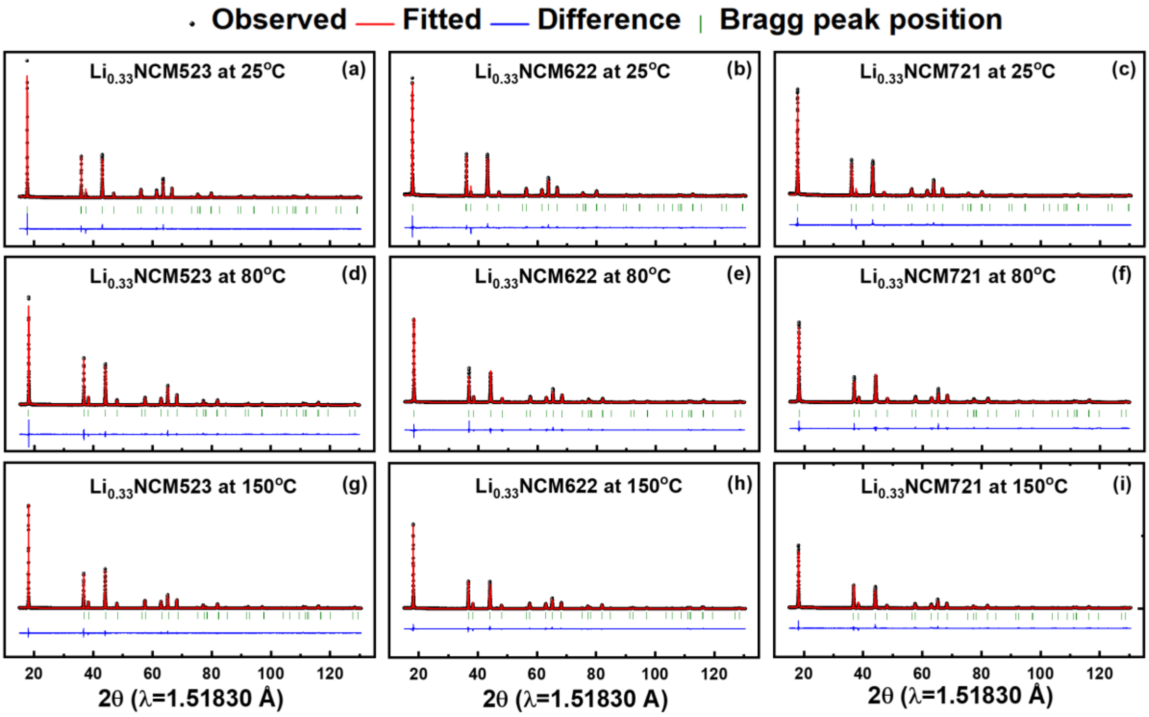

Figure S2. The HRPD patterns and Rietveld refinement results. The HRPD patterns (black dots) and Rietveld refinement results (red lines) of $Li_{0.33}Ni_{0.5+x}Co_{0.2}Mn_{0.3-x}O_2$ (x=0, 0.1, 0.2) cathode materials cooled to room temperature after heating up to 25□ (a-c), 80□ (d-f), and 150□ (g-i).

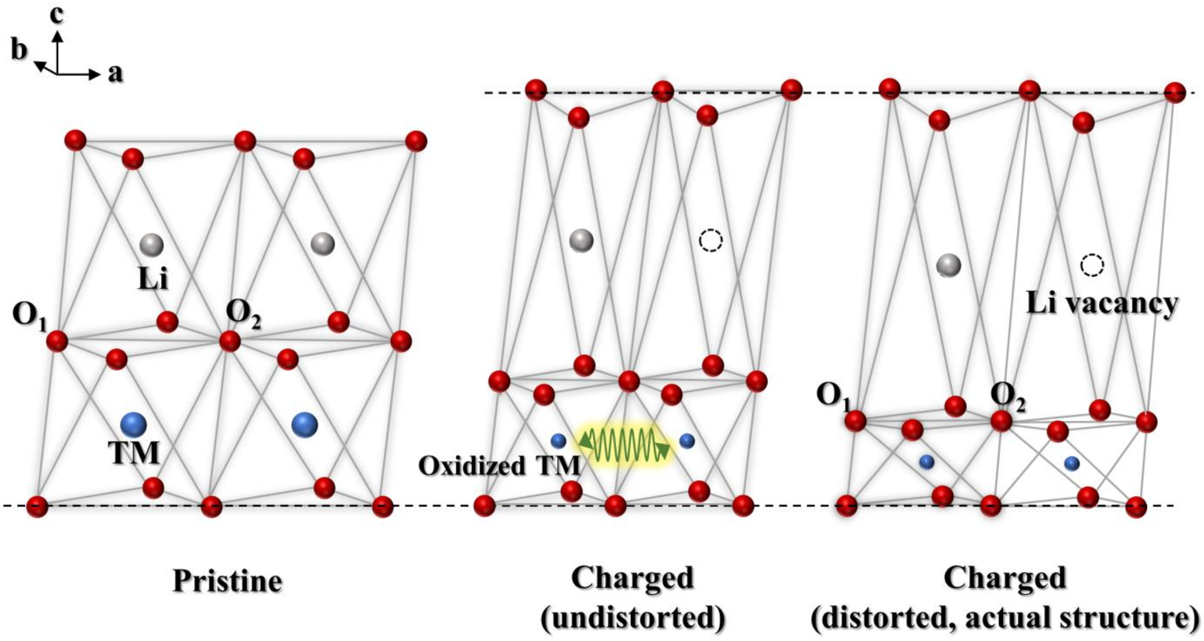

Figure S3. The schematic of TMO_6 and LiO_6 octahedron in the pristine and charged cathode material is based on the reference (pristine)[1] and result as shown in Table S1 and S2 (charged). The schematic on the charged state is exaggerated than the real structure to better represent the distortion of TMO_6 by the oxidation of TM ions. The TMO_6 octahedron in the real structure of the charged sample based on the Table S1 and S2 is larger in the c-axis direction than the actual structure in the above schematic, and the LiO_6 octahedron is smaller in the c-axis direction.

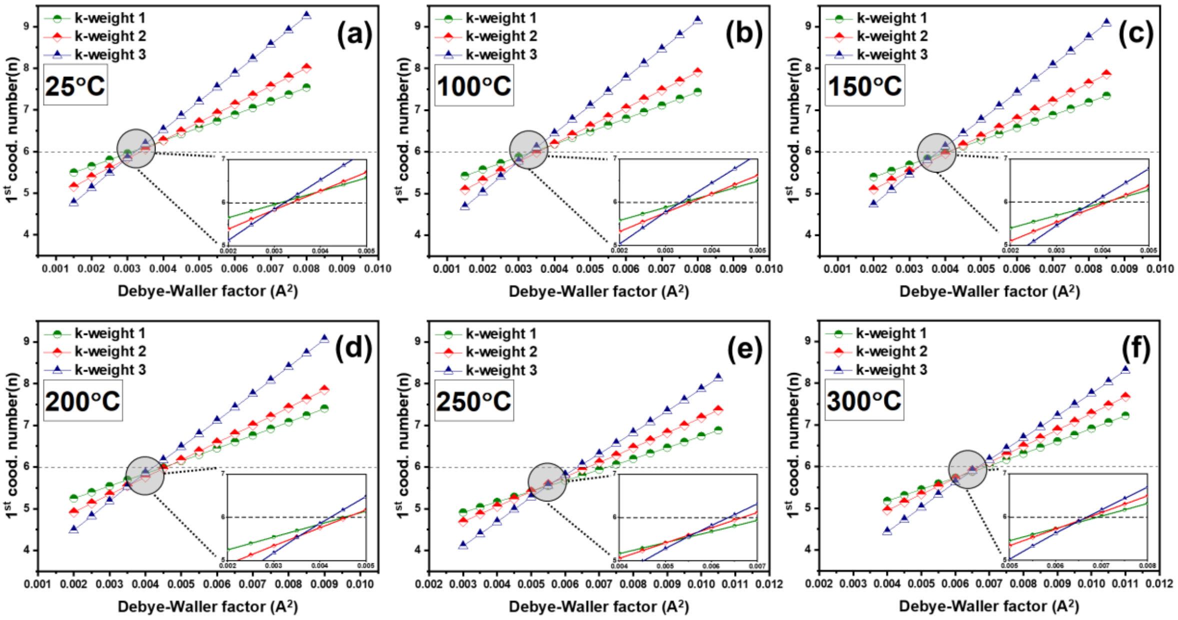

Figure S4. The k-weight (k = 1, 2, 3) dependent fitting of the first shell; Ni – O in NCM523 as applied temperature (a) – (f). The results were obtained by changing Debye-Waller factor, which is independent on the k-weight of EXAFS spectra. The intersection point indicates calibrated coordination number and Debye-Waller factor of first scattering shell. The same method was also applied to the fitting results of Co and Mn K-edge.

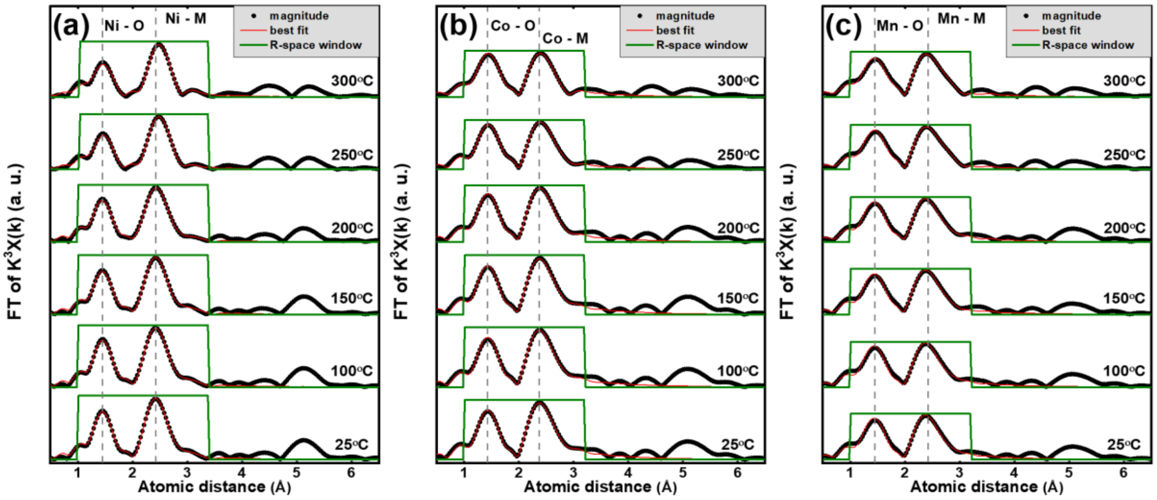

NCM523	Reduction factor	R-factor	Fitting range	K-range
Ni K-edge	0.75173	0.0014%	1 – 3.4Å	2 – 12.8k
Co K-edge	0.72132	0.0031%	1 – 3.2Å	2 – 10.8k
Mn K-edge	0.72709	0.0101%	1 – 3.2Å	2 – 10.8k

Figure S5. (a) – (c) show Fourier transform magnitude of Ni, Co, and Mn K-edge EXAFS spectra along with the fitted curve. The EXAFS spectra in R range over second scattering shell was fitted to get more accurate value, although we focus on the information of first scattering shell. The all fitted inner shell potential shift (ΔE) values in Ni, Co, and Mn K-edge were in the range of -8.5 to 0.0.

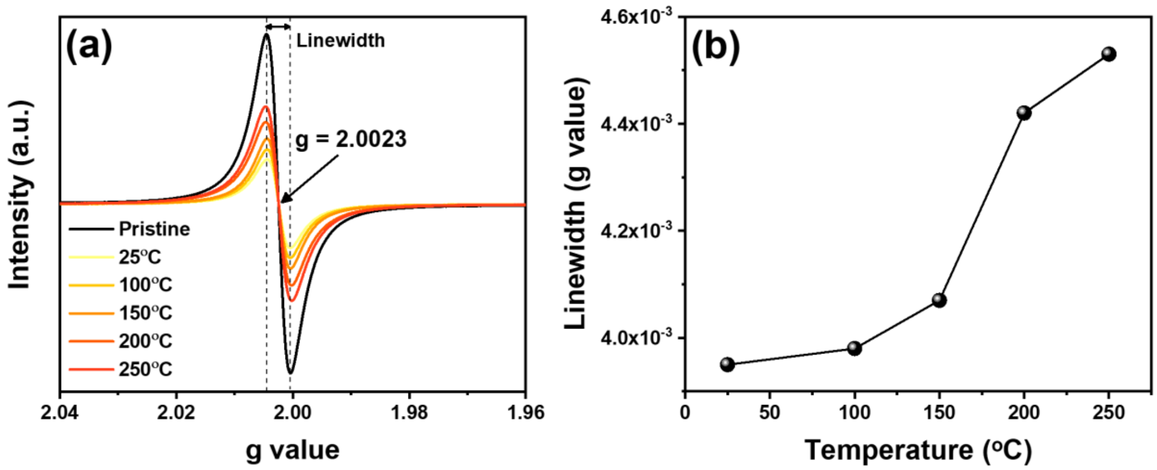

Figure S6. X-band EPR spectra on the pristine, nonheated, and heated $Li_{0.33}$NCM523 measured at 288 K (a), and the peak to peak linewidths of EPR signals (b) at each heated temperature. The microwave power and modulation amplitude were respectively set to 1 mW and 5 G. The weights of samples used in the EPR experiment were all set to about 0.02g.

The Figure S6 is electronic paramagnetic resonance (EPR) spectra on the pristine, nonheated, and heated $Li_{0.33}$NCM523 cathodes, measured at 288 K. The frequencies of ejected microwave were slightly different to optimize experimental condition according to the sample conditions (~ 0.9438GHz). The Figure S6 was plotted on the x-axis as the g value. The magnetization and net moment of charged layered cathode decrease as the state of charge of layered cathode material increases.[2-4] In our result, the charge process decreases the number of unpaired electron ($Ni^{2+/3+}$: $3d^8$ $t_{2g}{}^6e_g{}^2$ / $3d^7$ $t_{2g}{}^6e_g{}^1$ → Ni^{4+} : $3d^6$ $t_{2g}{}^6e_g{}^0$), which decreases the EPR signal intensity of charged sample than that of pristine sample. As the heated temperature increases, the reduction of Ni^{4+} ion occurs and the EPR intensity of charged sample increases. The reduction of Ni^{4+} is closely related to the formation of oxygen vacancies through charge compensation of oxygen electron to Ni^{4+} ion.[5] This relationship can be confirmed from the co-occurrence of reduction of Ni and formation of oxygen vacancies as shown in the Figure 5 and 7(a). The reduction of Ni^{4+} accompanied with oxygen vacancies formation increases the number of unpaired electrons, which is closely related to the increase of EPR signal intensity. Additionally, the peak to peak linewidth of EPR spectra can be

broadened by the random magnetic field from the random distribution of oxygen vacancies, so the EPR linewidth can be an indicator on the oxygen vacancies formation.[6,7] The EPR linewidth in our EPR results gradually increases in the heated temperature range from 25 ℃ to 250 ℃, which is consistent with the tendency of oxygen vacancies formation as shown in Figure 7(a). Therefore, we strongly believe that the increases of EPR intensity and linewidth in the Figure S6 can sufficiently support the reduction of Ni and oxygen vacancy formation due to temperature increase as shown in the Figure 5 and 7 (a).

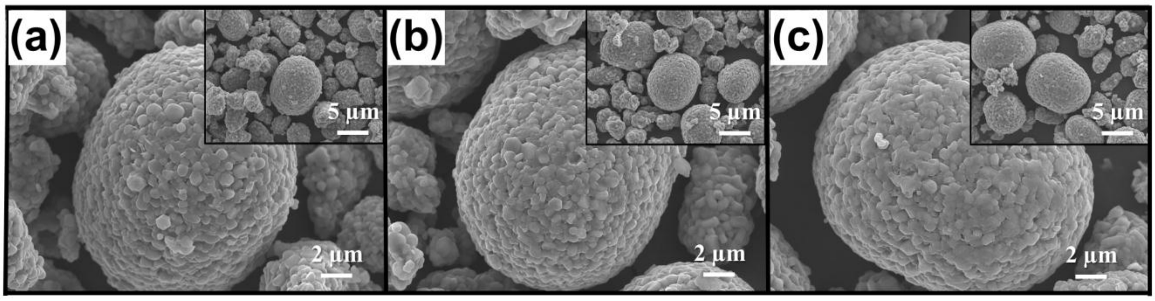

Figure S7. SEM images of as-prepared $LiNi_{0.5+x}Co_{0.2}Mn_{0.3-x}O_2$ (x=0, 0.1, 0.2) powders: (a) x = 0.0, (b) x = 0.1, and (c) x = 0.2.

Table S1. Rietveld refinement results of HRPD data for charged $Li_{0.33}Ni_{0.5+x}Co_{0.2}Mn_{0.3-x}O_2$ (x=0, 0.1, 0.2) cooled to room temperature after heating up to 25℃, 80℃, and 150℃. HRPD experiment was carried out in the range of 15 ~ 130° in 2θ (λ = 1.51830 Å). The Rietveld refinement executed by placing Li at 3b site, TM at 3a site, and oxygen at 6c site in the hexagonal structure with $R\overline{3}m$ space group. The $a_{hex.}$ and $c_{hex.}$ are lattice parameter of a- and c-axes, and Z_{ox} is atomic coordinate for oxygen ions. The V indicates the unit cell volume, and the S is the goodness-of-fit parameter. The B_{iso} is the isotropic displacement parameter, and the B_{iso} of TM and oxygen were fixed at 0.7 Å^2 in order to minimize the errors from the measurement temperature. The B_{iso} of Li at 3b site was fixed at 1.0 Å^2. The slab distances were calculated from the following equations; $D_{Li} = C_{hex.}(2Z_{ox}-1/3)$, $D_{TM} = 2C_{hex.}(1/3-Z_{ox})$.

At 25℃	$a_{hex.}$(Å)	$c_{hex.}$(Å)	V(Å^3)	Z_{ox}	TM slab (Å)	Li slab (Å)	R_p	R_{wp}	S(R_{wp}/R_{exp})
NCM523	2.81903(2)	14.4856(2)	99.69	0.2651(2)	1.976(2)	2.851(1)	6.16	8.59	1.814
NCM622	2.81664(1)	14.4682(2)	99.40	0.2658(4)	1.954(4)	2.868(2)	5.72	8.21	1.728
NCM721	2.81362(1)	14.4437(1)	99.02	0.2666(5)	1.927(5)	2.886(3)	5.85	8.08	1.874

At 80℃	$a_{hex.}$(Å)	$c_{hex.}$(Å)	V(Å^3)	Z_{ox}	TM slab (Å)	Li slab (Å)	R_p	R_{wp}	S(R_{wp}/R_{exp})
NCM523	2.81975(1)	14.4904(3)	99.77	0.2648(3)	1.986(3)	2.843(2)	12.1	15.9	1.558
NCM622	2.81787(2)	14.4737(2)	99.53	0.2654(3)	1.966(3)	2.858(2)	12.4	16.2	1.603
NCM721	2.81458(4)	14.4489(4)	99.14	0.2660(6)	1.945(6)	2.870(4)	13.1	18.7	1.968

At 150℃	$a_{hex.}$(Å)	$c_{hex.}$(Å)	V(Å^3)	Z_{ox}	TM slab (Å)	Li slab (Å)	R_p	R_{wp}	S(R_{wp}/R_{exp})
NCM523	2.82176(1)	14.4975(2)	99.96	0.2645(2)	1.995(2)	2.836(1)	10.7	14.5	1.557
NCM622	2.82009(2)	14.4750(2)	99.69	0.2650(2)	1.978(2)	2.846(1)	10.5	14.5	1.621
NCM721	2.81758(2)	14.4544(4)	99.37	0.2656(4)	1.958(4)	2.860(2)	10.3	14.5	1.699

Table S2. The length and angle in octahedron are obtained based on Rietveld refinement results of HRPD as shown in Table S1. The schematic based on these results is shown as Figure S3. The increase in oxidation state of TM ion, as the Ni content increases, causes stronger TM – O bond, which shifts the oxygen ion in the direction of F_{tot} by strongly pulling the oxygen toward the TM ion. The interaction is supported by the increase of angle of O_1 – TM – O_2 as increase Ni content. As the result, the size of Li slab in NCM721 is largest among $Li_{0.33}Ni_{0.5+x}Co_{0.2}Mn_{0.3-x}O_2$ (x=0, 0.1, 0.2) cathode materials.

Non-heated	Length of TM-O (Å)	Length of Li-O (Å)	Angle of O_1-Li-O_2 (°, degrees)	Angle of O_1-TM-O_2 (°, degrees)
$Li_{0.33}$NCM523	1.904(2)	2.163(2)	81.29(9)	95.50(6)
$Li_{0.33}$NCM622	1.897(3)	2.168(4)	81.00(5)	95.86(4)
$Li_{0.33}$NCM721	1.888(3)	2.173(4)	80.68(7)	96.28(3)

References

[1] W. Lee, S. Muhammad, T. Kim, H. Kim, E. Lee, M. Jeong, S. Son, J.-H. Ryou, W.-S. Yoon, *Adv. Energy Mater.* **2018**, *8*, 1701788.

[2] J. Reed, G. Ceder, Electrochem. *Solid‐ State Lett.* **2002**, *5*, 145.

[3] S. Miao, M. Kocher, P. Rez, B. Fultz, Y. Ozawa, R. Yazami, C. C. Ahn, *J. Phys. Chem. B* **2005**, *109*, 23473.

[4] N. A. Chernova, M. Ma, J. Xiao, M. S. Whittingham, J. Breger, C. P. Grey, *Chem. Mater*. **2007**, *19*, 4682.

[5] D. Qian, B. Xu, M. Chi, Y. S. Meng, *Phys. Chem. Chem. Phys*. **2014**, *16*, 14665.

[6] T. Matsuoka, L. S. Vlasenko, M. P. Vlasenko, T. Sekiguchi, K. M. Itoh, *Appl. Phys. Lett.* **2012**, *100*, 152107.

[7] S. Yildirimcan, K. Ocakoglu, S. Erat, F. M. Emen, S. Repp, E. Erdem, *RSC Adv.* **2016**, *6*, 39511.
